# Detecting Keratoconus in Adolescents with Anterior Segment Optical Coherence Tomography

**DOI:** 10.1155/2024/6655217

**Published:** 2024-06-07

**Authors:** Burcu Yücekul, Anika Förster, H. Burkhard Dick, Suphi Taneri

**Affiliations:** ^1^Haseki Training and Research Hospital, Department of Ophthalmology, Istanbul, Türkiye; ^2^Zentrum für Refraktive Chirurgie, Augenzentrum am St. Franziskus Hospital, Münster, Germany; ^3^Ruhr University Bochum, Eye Clinic, Bochum, Germany

## Abstract

**Purpose:**

Assessing the applicability of an algorithm developed for keratoconus detection in adolescents. This algorithm relies on optical coherence tomography (OCT) and incorporates features related to corneal pachymetric and epithelial thickness alterations.

**Methods:**

We retrospectively reviewed charts of patients under the age of 18 and divided them into four groups according to the Belin-Ambrosio display (Pentacam): normal, manifest, and subclinical keratoconus, as well as very asymmetric eye with normal topography and tomography (VAE-NTT). Corneal and epithelial thickness maps (Cirrus 5000 HD-OCT, Carl Zeiss Meditec, Germany) were evaluated by a human grader. In the first step, if at least one of four parameters (pachymetry minimum (pachy min), pachy minimum-median (min-med), pachy superonasal-inferotemporal (SN-IT), or epithelial (epi SN-IT)) exceeded its cut-off value, the eye was considered as suspect. In the second step, the combined presence of coincident thinning of total cornea and epithelium as well as concentric epithelial thinning lead to the diagnosis of keratoconus. Receiver operating characteristic (ROC) curves were generated to determine area under the curve (AUC), sensitivity, and specificity for the parameters.

**Results:**

The study involved 19 pediatric patients diagnosed with keratoconus, comprising 29 manifest keratoconic eyes, 3 eyes with subclinical keratoconus, and 5 VAE-NTT eyes. In addition, 22 eyes from 11 normal adolescents were included in the analysis. The AUC values of parameters in step 1 were 0.889 for pachy min, 0.997 for pachy min-med, 0.893 for pachy SN-IT, and 0.998 for epi SN-IT. When both steps were performed, this algorithm captured all manifest and subclinical pediatric keratoconic eyes. When all eyes of the keratoconus patients were combined, step 1 had 97.3% sensitivity and step 2 had 100% specificity.

**Conclusion:**

Using this OCT-based approach in adolescents yielded a high level of agreement with the current gold standard, tomography. Using them together, potentially also with other examinations may improve the diagnostic accuracy of KC in the pediatric population. Integration of this approach into the software of the device to facilitate automated evaluations is desired.

## 1. Introduction

Keratoconus is a progressive corneal ectasia that usually begins in adolescence. Typically, it has a faster progression leading to a poorer visual outcome in younger patients and may have severe consequences [1]. Therefore, especially in adolescents, it is important to diagnose the disease early and prevent its progression.

For many years, placido-based corneal topography was the main imaging modality for evaluating corneal surface curvature [2–4]. Today, Scheimpflug imaging has become the most commonly used imaging method in the diagnosis of keratoconus (KC), as it provides the opportunity to evaluate not only the anterior but also the posterior surface curvature and elevation, respectively, and also of corneal pachymetry [5–8]. The Global Consensus on Keratoconus and Ectatic Diseases stated that the best method for early and subclinical keratoconus diagnosis is tomography (e.g., Scheimpflug or optical coherence tomography) [9]. In recent years, studies based on anterior segment optical coherence tomography (AS-OCT) for the detection of KC have been published as AS-OCT provides both corneal pachymetric and epithelial thickness maps [10]. However, all of these studies examined the corneal pachymetric and epithelial thickness maps at different stages of KC in adult patients only [11–13]. In one of these studies, with a two-step decision algorithm, corneal pachymetric and epithelial thickness measurements and map patterns were evaluated and it was emphasized that this method has high specificity and sensitivity to detect keratoconus in patients over 18 years of age [10]. In a previous study, the value of this approach, which was modified for use with the Zeiss Cirrus OCT (Carl Zeiss Meditec, Dublin, CA, USA) was verified for adult patients [14]. However, to the best of our knowledge, although it is known that keratoconus often begins in adolescence, there is no study evaluating the use of corneal and epithelial thickness maps for keratoconus diagnosis in pediatric patients.

This study aimed to show the characteristics of corneal pachymetric and epithelial thickness maps and the validity of the two-step decision algorithm approach in pediatric patients that were previously published for adults [14].

## 2. Methods

### 2.1. Subjects

This retrospective study was conducted in a tertiary care clinic and followed the Declaration of Helsinki. The parents gave informed consent to the anonymous collection of their data for scientific analysis. Given the retrospective nature of this study, ethics committee approval was not required (Ethik Kommission Westfalen-Lippe 2019-214-f-S).

The medical data of patients under the age of 18 who presented at St. Francis Hospital Münster, Center for Refractive Surgery between February 2020 and September 2021 were reviewed. The diagnosis of keratoconus was established by the combination of corrected distance visual acuity (CDVA), slit-lamp findings, and the Belin/Ambrósio Enhanced Ectasia Display (BAD) integrated into the Pentacam AXL (OCULUS Optikgeräte GmbH, Wetzlar, Germany). Keratoconic eyes were divided into two subgroups according to the following criteria: (1) Manifest KC: slit-lamp findings associated with keratoconus (Vogt striae, Fleischer ring, Munson sign, Rizzuti sign, and apparent focal corneal bulging and thinning), and/or had a red color-coded number (at least 2.6 standard deviation from the mean) in the BAD Deviation (D) value. [15] (2) Subclinical KC: CDVA equal to or better than 20/20; no slit-lamp findings of keratoconus and yellow color-coded number in the BAD-D value (≥1.6, to <2.6). The fellow eye of a keratoconic eye which had a white color-coded number (<1.6 standard deviation from the mean) in the BAD-D value was assigned to very asymmetric eye with normal topography and tomography (VAE-NTT) [15].

Patients who were referred to St. Francis Hospital Münster, Center for Refractive Surgery with the suspicion of keratoconus and did not have any ophthalmic disease other than refractive error or amblyopia and had normal topography and tomography parameters on Pentacam including BAD-D values <1.6, were considered as control group.

Patients with a history of ophthalmic surgery and recent contact lens usage (soft contact lens within 2 weeks or rigid gas permeable lens within 4 weeks) were excluded. Also, keratoconic eyes with corneal scarring whose optical coherence tomography (OCT) scans had very irregular corneal and epithelial thickness patterns were excluded from the data analysis.

## 3. Scheimpflug Tomography

Pentacam AXL (OCULUS Optikgeräte GmbH, Wetzlar, Germany) was used to evaluate the corneal tomography. The final D readings of the Belin/Ambrósio Enhanced Ectasia Display were acquired from the outputs of this device to classify the study groups.

### 3.1. Optical Coherence Tomography

Corneal thickness (CT) and corneal epithelial thickness (ET) maps were obtained from a Spectral-Domain Cirrus 5000 HD-OCT (Carl Zeiss Meditec, Germany) with a corneal adaptor lens. A color-coded thickness map of the cornea and the epithelium is obtained by 24 radial B-scan lines with a scan depth of 2.0 mm. Each B-scan is composed of 1024 A-scans.

Cirrus HD-OCT review software (version 11.5) processed the data and provided three concentric ring-shaped zones centered on the center of the cornea (central: 0–2 mm, paracentral: 2–5 mm, and mid-peripheral: 5–7 mm) for the average automated CT, as well as four concentric zones for ET (central: 0–2 mm, paracentral: 2–5 mm, mid-peripheral: 5–7 mm, and peripheral: 7–9 mm). The color scale of the CT map was constant in 20 *μ*m steps and the ET map was constant in 1 *μ*m steps.

The software algorithm measures CT as the distance between the air-tear and cornea-aqueous interfaces and it includes epithelium. It measures ET as the distance between two hyperreflective lines on B-scans that correspond to stromal surface and anterior surface of the Bowman layer. An important difference of this software from the ones integrated in other OCT devices is that it minimizes the inclusion of the tear film in the measurement of ET.

### 3.2. Two-Step Decision Algorithm

**Figure 1:**
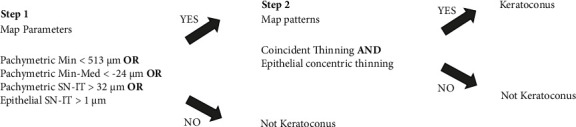
Two-step decision algorithm for the detection of keratoconus.

Using an Avanti OCT (Optovue, Fremont, CA, USA) a two-step decision algorithm was originally described to identify a keratoconic cornea by Yang et al. [10]. We modified their approach for use with the Zeiss Cirrus OCT by changing same parameters and their respective cut-off values and validated it in adult patients [14] (Figure 1).

In step 1, four quantitative parameters that were shown on the outputs of the Cirrus OCT device were used. These parameters were: (1) Pachymetric (Pachy) minimum (min): minimum corneal thickness (2) Pachy minimum-median (min-med): the minimum corneal thickness minus the median corneal thickness. (3) Pachy superonasal-inferotemporal (SN-IT): the average corneal thickness of the superonasal octant minus the average corneal thickness of the inferotemporal octant between the 2 mm and 5 mm diameter rings. (4) Epithelial (Epi) superonasal-inferotemporal (SN-IT): the average epithelial thickness of the superonasal octant minus the average epithelial thickness of the inferotemporal octant between the 2 mm and 5 mm diameter rings. Loureiro et al. showed that the pachymetric and epithelial thickness measurements by Zeiss OCT in the healthy children were similar with the measurements of adults [16]. Due to the low number of control patients in our study and the previous study showing that child and adult patient measurements were similar, we used the cut-off values that we obtained from the 25th percentile (pachymetric minimum and min-med) or the 75th percentile (pachymetric SN–IT and epithelial SN-IT) measurements in the control group of adult patients in the previous study. The cut-off values were: Pachy min < 513 *μ*m, pachy min–med < −24 *μ*m, pachy SN–IT > 32 *μ*m, and epi SN–IT >1 *μ*m. If at least one of these parameters exceeded its cut-off value, then the eye was considered as suspect KC and proceeded to the second step of the decision algorithm. If none of these four parameters exceeded the cutoff values, the eye was accepted as normal.

**Figure 2:**
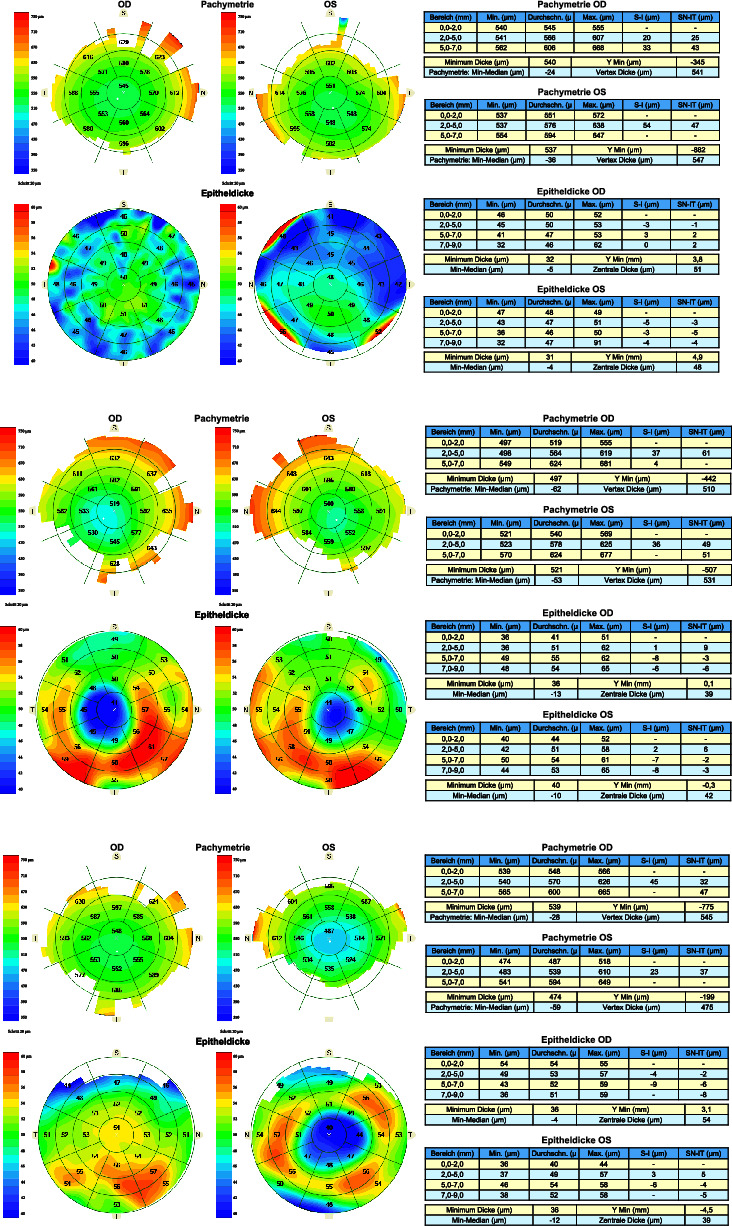
(a) Corneal and epithelial thickness maps of a normal patient (control group). (b) Corneal and epithelial thickness maps of a patient with bilateral manifest keratoconus. (c) Corneal and epithelial thickness maps of a patient with a normal right and a keratoconic left eye according to the algorithm in agreement with Belin/Ambrosio display from the Pentacam.

In step 2, a human grader (B. Y.) evaluated the presence of coincident thinning of total cornea and epithelium as well as of concentric epithelial thinning by examining the images of CT and ET map patterns. Coincident thinning was noted if the thinnest pachymetric point within the analytical zones and the thinnest epithelial point were in the same or adjacent zone. An epithelial concentric thinning pattern was noted if there was at least a 5 color-scale step change (every 1 *μ*m change in epithelial thickness corresponds to a step on the color scale) in the epithelial thickness map together with 1 complete ring around the thinnest epithelial point in the analytical zones. Sample study population images (KC patients and controls) are shown in Figure 2.

### 3.3. Statistical Analysis

All data analyses were performed using SPSS package program version 25.0 (SPSS Inc., Chicago, IL, USA). In descriptive statistics, categorical variables were given as mean and standard deviation values. Qualitative variables were presented as numbers and percentages. One Way ANOVA test was used for the comparison of numerical variables when the normal distribution condition was met in the groups, and Kruskal–Wallis test was used when the normal distribution condition was not met. Tukey's post hoc test was used for subgroup analysis. Receiver operating characteristic (ROC) curve analyses were performed to assess the diagnostic accuracy of the four quantitative OCT parameters, first and second step evaluation. A *P* value less than 0.05 for all tests was considered statistically significant.

## 4. Results

The study involved 19 pediatric patients diagnosed with keratoconus, comprising 29 manifest keratoconic eyes, 3 eyes with subclinical keratoconus, and 5 VAE-NTT eyes. One eye of the KC patients was excluded from the study due to severe corneal scar formation. The control group was comprised of 22 eyes of 11 age-matched patients that had no ocular disease other than refractive errors or amblyopia.

The demographic characteristics of the study groups are given in Table 1. There was no statistically significant difference in sphere values, but subclinical and VAE-NTT groups had lower cylinder values than the control and manifest KC groups (*p* < 0.05 for all of them). The CDVA values in the KC group were significantly lower than in the control and VAE-NTT groups.

**Figure 3:**
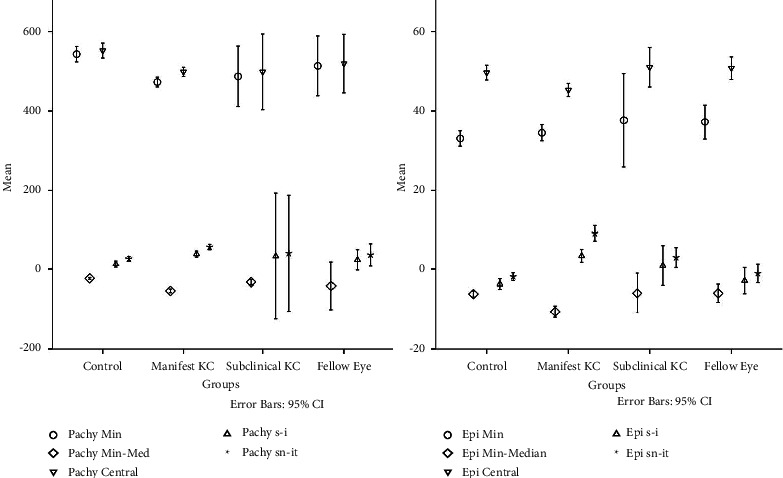
Pachymetric and epithelial thickness variations between groups (in *μ*m).

Corneal pachymetric and epithelial thickness variations between groups are shown in Figure 3.

There was a statistically significant difference in minimum pachy, pachy min-med, central pachy, pachy S-I, and pachy SN-IT between KC and control groups (*p* < 0.001 for all of them). In the subclinical KC group, there was a significant difference in only pachy min-med values from those of the control group (*p* < 0.001). Considering epithelial map parameters, only epi SN-IT had a significant difference in both subclinical and manifest KC groups compared to the control group (*p*=0.006; *p* < 0.001, respectively). Epi SN-IT was also one of the parameters that we used in step 1 of the decision algorithm approach.

**Figure 4:**
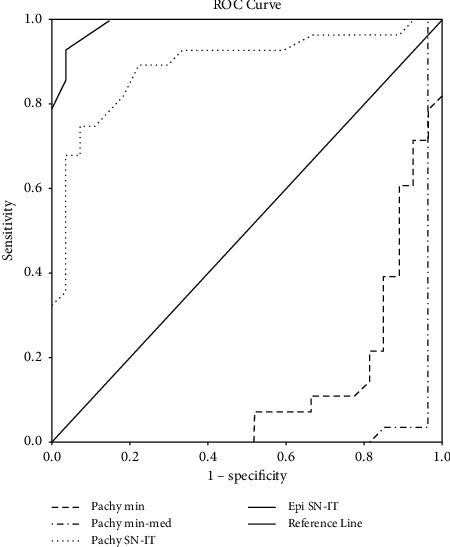
Receiver operating characteristic curves illustrating the diagnostic ability of step 1 parameters measured by anterior segment optical coherence tomography in discriminating keratoconus from controls.

The sensitivity and specificity of four individual OCT parameters are shown in Table 2, and Figure 4 shows the ROC curves of the parameters that were used in step 1.

For these comparisons, manifest, subclinical keratoconus, and VAE-NTT groups were compared with the control group. According to this comparison epi SN-IT had 95% specificity and 100% sensitivity. Map pattern characteristics of the patient groups are shown in Table 3.

Manifest KC patients had more pachy and epi color steps than control patients (*p* < 0.001, for both). Pachy concentric thinning was observed in all patients. In contrast, epi concentric thinning was observed in only 9% of the control group, 60% of the VAE-NTT group, but in all the keratoconic eyes.

In step 1 of the decision algorithm, 68% of the control eyes were suspicious for KC, but step 2 correctly ruled out all of these (Table 4).

This two-step decision algorithm captured all manifest and subclinical pediatric KC patients. When these manifest and subclinical keratoconic eyes were combined and compared with the control group, step 1 had a high sensitivity (97.3%) and step 2 had 100% specificity (Table 5).

## 5. Discussion

In this study, we evaluated the applicability of a systematic evaluation of OCTs for KC diagnosis in a pediatric population which we had previously developed in adult patients [14]. Measurements obtained with the Zeiss Cirrus OCT were analyzed using a two-step decision algorithm approach consisting of four numeric OCT parameters and map pattern recognition criteria.

Keratoconus in children differs from KC in adults in several clinical features [17]. In contrast to KC in adults, pediatric keratoconus tends to show a more rapid progression [18]. In the pediatric population, KC can be diagnosed late due to the lack of symptoms, and sometimes acute corneal hydrops may be the presenting feature. Furthermore, the prognosis of penetrating keratoplasty is poorer in pediatric KC [19]. Therefore, early diagnosis of KC with reliable clinical tests is especially important in children.

Today, corneal pachymetry and epithelial thickness can be evaluated with AS-OCT, a rapid, noninvasive tool with excellent measurement repeatability [11, 19]. Epithelial remodeling may precede any changes in the anterior surface of the cornea, so epithelial evaluation may allow earlier diagnosis of KC [20]. Epithelial thickness assessments may increase the sensitivity and specificity of KC screening compared to corneal topography alone and may be useful in diagnosing KC in clinical practice. Such epithelial thickness changes in KC have been studied by other authors with different devices in adults [21, 22]. In one of these studies, central corneal thickness (CCT), stromal layer thickness, and central epithelial thickness were significantly different in eyes of adult keratoconus patients from the control group [23]. However, in the pediatric population, epithelial thickness maps have only been examined in healthy children with AS-OCT [16]. In this study, epithelial thickness map characteristics of pediatric patients belonging to different stages of KC were evaluated for the first time.

Many topographic and tomographic metrics have been utilized to detect KC. Ambrosio and associates reported a sensitivity of 99% and a specificity of 97.5% with the BAD-D index which is a combination of nine BAD-based tomographic parameters using regression analysis for discriminating keratoconic and normal eyes [24, 25]. Diagnosing early-stage keratoconus and subclinical KC, on the other hand, is challenging due to the lack of a precise definition in the literature. Many definitions have been used to describe the non-manifest stages of keratoconus, such as subclinical keratoconus, suspected keratoconus, and forme fruste keratoconus [25, 26]. Muftuoglu et al. showed in their study that the D index had the best specificity to differentiate between keratoconus and subclinical keratoconus eyes and control eyes [27]. Heidari et al. evaluated the corneal topographic, tomographic, and biomechanical indices for detecting clinical and subclinical keratoconus and they indicated that the AUC of BAD-D is 0.842 in differentiating subclinical KC from normal and 0.992 in manifest KC [5]. BAD-D was used as a reference in many keratoconus detection studies and high AUC values have been observed. [28, 29]. This study used highly sensitive, specific, and commonly used index for the classification of groups. The two-step decision algorithm approach accurately identified all manifest and subclinical keratoconic eyes in perfect accordance with BAD-D.

Many parameters have been evaluated to detect keratoconus with different AS-OCT devices in adults [11–13, 30]. In step 1 of the decision algorithm, four parameters were used. Pachymetric minimum and min-med indicate focal thinning of the cornea; pachymetric SN-IT indicates asymmetric localized corneal thinning typical of keratoconus; and epithelial SN-IT indicates asymmetric corneal epithelial thinning over the stromal cone. In this study, pachy min-med and epi SN-IT showed statistically significant differences in manifest and subclinical KC groups compared with the control group (*p* < 0.05 for all). Hashemi et al. evaluated the corneal thickness distribution patterns with AS-OCT and their results showed that CCT and pachy min-med are the most sensitive indices for the diagnosis of KC [31]. Similar to their results, in this study pachy min-med has the highest AUC after epi SN-IT.

The most challenging task is to distinguish clinically VAE-NTT fellow eyes of asymmetric KC patients from healthy eyes. In this study, 5 VAE-NTT patients were classified as normal according to the decision algorithm. Hwang et al. combined 13 parameters from Scheimpflug tomography and AS-OCT to discriminate control eyes from the clinically unaffected eyes with no physical findings on slit-lamp examination, no definitive abnormalities on corneal imaging, and corrected distance acuity of 20/20 or better from 30 patients with highly asymmetric KC eyes and they reached 100% sensitivity and specificity [32]. Two of these 13 parameters were pachy min and pachy min-med that were used in the first step of the decision algorithm of this study. Their findings demonstrated that it is difficult to diagnose KC correctly using individual metrics from Scheimpflug tomography or SD-OCT alone. They also demonstrated that the combination of metrics from both devices yielded a good discriminative power. In their study, in contrast to this study, there was a statistically significant difference between the BAD-D values of clinically normal fellow eyes and the BAD-D values of the control group. In contrast, there was no statistically significant difference between the BAD-D values of VAE-NTT group and the control group (*p* values <0.001 and =1.0, respectively). That might be the reason that the decision algorithm could not capture these eyes. On the other hand, their results suggest that adding machine-derived metrics from Scheimpflug imaging to a decision algorithm OCT approach might yield improved diagnostic accuracy. On the other hand, BAD-D had AUC of 0.839 between control and very asymmetric ectasia with normal topography eyes [29].

In step 2 of the decision algorithm, coincident thinning and epithelial concentric thinning were seen in 100% of the manifest and subclinical keratoconic eyes of children. In the study of Yang et al. [10], an SD-OCT system (Avanti, OptoVue Inc.) was used, and coincident and epithelial concentric thinning were seen together in 97.8% of manifest keratoconic and 100% of subclinical keratoconic eyes of adults. The fact that the tear film layer is (almost) not included in the epithelial thickness measurements of the Zeiss device may be regarded as an advantage compared to the Avanti OCT [33].

The primary limitations of this study are its retrospective design, and lack of follow-up. If VAE-NTT had been followed up, manifest KC might have been seen later. We acknowledge that the small sample size may prevent the study from being more robust; nevertheless, this is the first study using AS-OCT in the diagnosis of pediatric keratoconus patients. Furthermore, some improvements in the decision algorithm may occur if it could be implemented in future versions of the Zeiss Cirrus 5000 HD-OCT software for automated evaluation. For instance, automatic quantitative measurement of the distance between the thinnest points of pachymetric and epithelial maps may be more accurate than using the “same or adjacent map region” criteria by a human grader. Similarly, the assessment of concentric epithelial thinning patterns by a human grader in the current study could be automated for better reliability in the future.

In conclusion, our findings demonstrated that pachymetric and epithelial map parameters and patterns from the Zeiss Cirrus OCT can be used in the detection of keratoconus in a pediatric population. Further improvements in the software of the device to facilitate automated evaluations are desired. Using this OCT-based approach together with tomography and other examinations may improve the diagnostic accuracy of KC in the pediatric population even further.

## Figures and Tables

**Table 1:** Demographic characteristics of study groups.

Variables	Control	Manifest KC	Subclinical KC	VAE-NTT	*P* value
Age	15.3 ± 2.3	15.9 ± 1.1	15.3 ± 1.5	15.8 ± 1.3	0.922
Sphere (D)	−0.07 ± 2.57	0.17 ± 1.55	−0.83 ± 1.04	0.10 ± 0.54	0.388
Cylinder (D)	−1.71 ± 1.38	−2.60 ± 1.97	−0.08 ± 0.14	−0.50 ± 0.39	0.005
CDVA (Snellen)	1.1 ± 0.3	0.8 ± 0.3	1.2 ± 0.3	1.4 ± 0.1	0.002

KC = keratoconus; CDVA = corrected distance visual acuity; VAE-NTT = very asymmetric eye with normal topography and tomography; the values are given mean ± standard deviation (SD); ANOVA test was used for the comparison of subgroups.

**Table 2:** Sensitivity and specificity of step 1 parameters in discriminating keratoconus from controls.

	Pachymetric min	Pachymetric min-med	Pachymetric SN-IT	Epithelial SN-IT
Sensitivity (%)	73	76	86	100
Specificity (%)	90	100	71	95
AUC	0.889	0.997	0.893	0.998
PPV (%)	83	94	82	97
NPV (%)	70	76	62	84

min = minimum thickness; min-med = minimum-median thickness; SN-IT = superonasal-inferotemporal thickness; AUC = area under curve; PPV = positive predictive value; NPV = negative predictive value.

**Table 3:** Characteristics of pachymetric and epithelial OCT map patterns.

	Pachy color steps	Epi color steps	Pachy concentric thinning, eyes, *n* (%)	Epi concentric thinning, eyes, *n* (%)	Coincident thinning, eyes, *n* (%)
Control	2.9 ± 0.6	6.5 ± 1.5	22 (100)	2 (9.0)	0 (0)
Manifest KC	5.0 ± 1.5	12.0 ± 2.1	29 (100)	29 (100)	29 (100)
Subclinical KC	3.3 ± 0.5	8.3 ± 2.5	3 (100)	3 (100)	3 (100)
VAE-NTT	3.2 ± 1.6	7.8 ± 4.1	5 (100)	3 (60)	0 (0)
*P* values^*∗*^	<0.001	<0.001			

OCT = optic coherence tomography; pachy = pachymetry; epi = epithelial; KC = keratoconus; VAE-NTT = very asymmetric eye with normal topography and tomography; ^*∗*^ = comparison for manifest KC and control groups.

**Table 4:** Results of two-step decision tree.

	Step 1, suspicious for KC eyes, *n* (%)	Step 2, KC eyes, *n* (%)
Control	15 (68)	0 (0)
Manifest KC	29 (100)	29 (100)
Subclinical KC	3 (100)	3 (100)
VAE-NTT	4 (80)	0 (0)

KC = keratoconus; VAE-NTT = very asymmetric eye with normal topography and tomography.

**Table 5:** Diagnostic value of two-step decision tree.

	Step 1 (%)	Step 2 (%)
Sensitivity	97.3	86.5
Specificity	31.8	100

## Data Availability

The data used to support the findings of this study are made available from the corresponding author upon reasonable request.
